# Nosocomial Infection with Vancomycin-dependent Enterococci^1^

**DOI:** 10.3201/eid1007.030993

**Published:** 2004-07

**Authors:** Paul A. Tambyah, John A. Marx, Dennis G. Maki

**Affiliations:** * University of Wisconsin Medical School, Madison, Wisconsin, USA

**Keywords:** Vancomycin, antibiotic use, antimicrobial resistance, Enterococcus, nutritionally deficient microorganisms, vancomycin-resistant enterococci (VRE), vancomycin-dependent enterococci (VDE), dispatch

## Abstract

We report three patients infected with unique strains of vancomycin-dependent enterococci. Two were first infected by genetically identical strains of vancomycin-resistant enterococci (VRE). All three patients had much greater exposure to vancomycin and third-generation cephalosporins than did two control groups (patients infected with VRE and hospitalized patients without enterococcal infections). While antimicrobial pressure promotes nosocomial colonization by VRE, prolonged exposure to vancomycin may foster the transition from vancomycin resistance to dependence.

## Abstract 2

In three patients with nosocomial vancomycin-resistant enterococcal infections, infections attributable to vancomycin-dependent enterococci developed.

Vancomycin-resistant enterococci (VRE) are major nosocomial pathogens worldwide (*1*). Recent case reports, however, describe nosocomial infections caused by enterococci that require vancomycin for growth (*2**–**12*). Since 1993, we have identified three patients in our center infected by vancomycin-dependent enterococci (VDE). We report the microbiologic features and molecular epidemiology of nosocomial infection caused by these organisms.

## Methods

Enterococci showing growth on media containing 6 µg/mL of vancomycin and an MIC >8 µg/mL were considered vancomycin-resistant. Strains unable to grow in the absence of vancomycin 6 µg/mL, despite multiple subcultures, were considered vancomycin-dependent.

The genotypic basis of vancomycin resistance was determined by using polymerase chain reaction to amplify sequences coding for resistance, using oligonucleotide primers for *vanA* (5´CATGAATAGAATAAAAGTTGC and 5´CTTATCACCCCTTTAACG, Department of Pharmacology, University of Wisconsin-Madison, Madison, WI) and *vanB* (5´AAATTCGATCCGCACTACATC and 5´AACGATGCCGCCATCCTCCTG, University of Wisconsin Biotechnology Center, Madison, WI). Susceptibility was assessed by National Committee for Clinical Laboratory Standards criteria using Mueller-Hinton II agar (BBL, Becton-Dickinson, Cockeysville, MD) containing vancomycin 6 µg/mL. The capacity of D-alanyl-D-alanine to support growth of the VDE was tested by using 2.5-µg and 5-µg disks of D-alanyl-D-alanine on Mueller-Hinton II agar. Screening for revertants was performed by plating serial dilutions of an overnight culture of VDE in vancomycin-containing broth to Mueller-Hinton II agar with and without vancomycin, which was incubated for 48 hours at 35°C. Molecular relation of strains was determined by using pulsed-field gel electrophoresis (PFGE) after digestion of genomic DNA with restriction endonuclease *Sma*1 (Gibco BRL, Promega, Madison, WI) (*13*).

## Case Reports

### Patient 1

**Figure 1:**
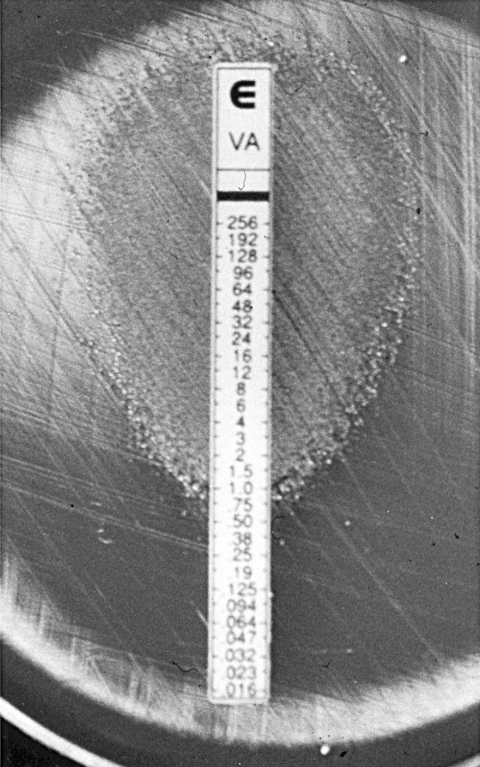
Etest (AB Biodisk, Solna) vancomycin susceptibility–testing strip on sheep-blood agar inoculated with vancomycin-dependent enterococci (VDE). VDE strain can only grow contiguous to the end of the strip with the highest concentrations of vancomycin. Isolated colonies are also growing far from the strip; they represent revertants to vancomycin independence.

A 32-year-old woman with long-standing type 1 diabetes mellitus and end-stage renal disease was admitted for a kidney-pancreas transplant. Postoperatively, she had multiple complications, including transplant renal failure and intraperitoneal infection caused by vancomycin-resistant *Enterococcus faecium*. She received vancomycin, teicoplanin, imipenem, amikacin, cefazolin, ceftazidime, ciprofloxacin, gentamicin, metronidazole, ticarcillin-clavulanate, trimethoprim-sulfamethoxazole, and intravenous amphotericin B. On hospital day 53, intraabdominal fluid specimens obtained at surgery yielded vancomycin-resistant *E. faecium* that did not grow on media without vancomycin (Figure 1). The infection was treated with surgical drainage and a combination of teicoplanin and gentamicin. Despite this, the patient died of refractory sepsis on hospital day 268. VDE were isolated from multiple intraabdominal cultures in the month before death.

### Patient 2

A 40-year-old woman with type 1 diabetes mellitus and end-stage renal disease received a kidney-pancreas transplant, which was complicated by multiple intraabdominal abscesses that were drained surgically. On posttransplant day 82, VRE were isolated from intraabdominal cultures. The transplanted kidney was removed on posttransplant day 115 and the transplanted pancreas 10 days later. However, the patient continued to show signs of sepsis. Blood cultures were positive on radiometric monitoring. Subculturing onto media containing vancomycin confirmed bacteremia with a strain of *E. faecium* that did not grow in the absence of vancomycin. The patient remained critically ill, despite prolonged therapy with intravenous quinupristin-dalfopristin, and died after 4 days of refractory VDE bacteremia, 132 days after transplantation.

### Patient 3

A 47-year-old woman with chronic myelogenous leukemia received a matched-unrelated donor bone marrow transplant. Subsequently, when severe graft-versus-host disease, acute renal failure, cyclosporine neurotoxicity, prolonged respiratory failure, and bacteremia with *Corynebacterium* spp. resistant to β-lactam antimicrobial agents developed, the patient received a prolonged course of vancomycin. On hospital day 80, when vancomycin-containing media was used, she was found to have catheter-associated urinary tract infection with a strain of enterococcus that required vancomycin for growth. Efforts were not made to eradicate VDE from the urine. The patient ultimately died of refractory graft-versus-host disease with multiple organ dysfunction syndrome on posttransplant day 87.

### Case-Control Study

Potential risk factors for nosocomial infection were compared in the 3 patients and 10 randomly selected patients with nosocomial infection caused by VRE and 10 at-risk, concurrently hospitalized patients not infected by enterococci. Controls were matched by age and admission to the same hospital service as patients in the VRE cohort.

## Results

**Figure 2:**
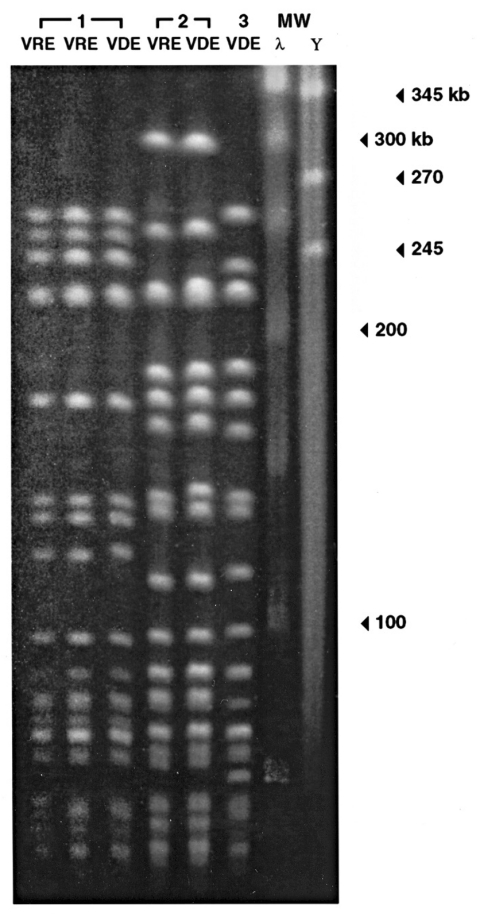
Pulsed-field gel electrophoresis of the three strains of vancomycin-dependent enterococci (VDE) and, in two cases, a vancomycin-resistant enterococci (VRE) strain isolated before the VDE in the same patient. The three VDE strains appear to be genetically distinct, although two may be related. In both cases in which VRE was isolated before VDE, VRE and subsequent VDE strains appear genetically identical. RFLP, restriction fragement length polymorphism; MW, molecular weight; λ, lambda ladder, Y, yeast chromosome marker.

PFGE analysis indicated that the three strains of vancomycin-dependent enterococci were clonally distinct (Figure 2) when the criteria of Tenover et al. were used (*14*). In the two cases in which strains of VRE were isolated before VDE were first detected, the restriction fragment patterns of the initial VRE strain and subsequent VDE isolate were identical.

All three strains of VDE were *E. faecium*; two were genotype *vanA*, and one was *vanB*. All showed resistance to penicillin, ampicillin, amoxicillin-clavulanate, gentamicin, and erythromycin. All were susceptible to quinupristin-dalfopristin; two were intermediately susceptible, and one was susceptible to teicoplanin. The rate of spontaneous reversion to nondependence on vancomycin was 1.2 x 10^–6^ for strain 1, 2.5 x 10^–6^ for strain 2, and 2.6 x 10^–3^ for strain 3. Growth of VDE was not supported by D-alanyl-D-alanine.

All three patients infected by VDE were female transplant recipients and experienced posttransplant acute renal failure; by contrast, the VRE group had lower exposure to the intensive care unit (Table). No other significant differences were noted between the two groups of patients with enterococcal infection and the group of at-risk controls in underlying conditions, severity of illness, or exposure to invasive devices. However, in the 60 days before onset of the nosocomial enterococcal infection, major differences in exposure to antimicrobial agents occurred: mean ± standard deviation (SD) total antimicrobial days 103 ± 40 for VDE, 86 ± 31 for VRE, and 28.6 ± 23.1 for noninfected controls (VDE or VRE vs. controls, p < 0.01), especially vancomycin (27 ± 14 days for VDE, 9 ± 10 for VRE, and 5.7 ± 7.6 for controls; VDE vs. VRE, p = 0.03) and third-generation cephalosporins (17.0 ± 11.4 days for VDE, 15.6 ± 11.9 for VRE, and 2.9 ± 4.8 for noninfected controls; VDE or VRE vs. controls, p < 0.01). All 3 patients with VDE infection died during hospitalization, contrasted with 3 of 10 patients infected with VRE and 2 of 10 uninfected control patients who died (p = 0.03).

**Table:** Clinical and epidemiologic features of patients with nosocomial enterococcal infections and uninfected control patients^a^

Features	VDE (n = 3)	VRE (n = 10)	Uninfected control patients (n = 10)
Age, y, mean ± SD	39.0±7.5	41.7±20.2	51.1±13.0
Sex, no.			
Male	0	5	7
Female	3	5	3
Duration of hospitalization, days, mean ± SD	41.7±13	33.6±12.1	37.6±44.7
ICU stay, days, mean ± SD	9.3±4.0	1.0±1.9^b^	7.4±7.4
Site of nosocomial enterococcal infection			
Primary bloodstream infection	0	5	0
Surgical wound infection	1	2	0
Intraabdominal infection	1	1	0
Urinary tract Infection	1	1	0
Service, no.			
Medicine or pediatrics	1	5	5
Surgery	2	5	5
Associated conditions, no.			
Malignancy	1	4	2
Diabetes mellitus	2	3	4
Renal failure	3	3	4
Trauma	0	2	0
Transplant recipient	3	4	2
APACHE II score, mean ± SD	18.7±2.1	15.1±6.7	19.4±10.0
Serum creatinine, mg/dL, mean ± SD	2.8±0.5^c^	1.5±0.8	1.9±1.8
Days administered antimicrobial agent			
Vancomycin	27.3±13.7^d^	9.1±10.3	5.7±7.6
Aminoglycosides	11.3±6.7	9.7±9.8	2.5±5.1
First- or second-generation cephalosporins	0.7±0.6	1.1±2.2	3.6±7.5
Third-generation cephalosporins	17.0±11.4^e^	15.6±11.9^f^	2.9±4.8
Quinolones	10.3±5.5	8.0±9.2	3.4±4.8
Clindamycin	2.3±4.0	7.1±11.7	1.2±3.8
Metronidazole	4.3±7.5	4.4±6.3	1.8±3.8
Trimethoprim-sulfamethoxazole	32.7±18.0^e^	14.6±19.2	2.7±5.7
Others	1.0±1.7	5.6±9.5	4.8±9.6
Total	106.3±44.7^e^	83.5±29.4^f^	28.6±23.1

^a^VDE, vancomycin-dependent enterococci; VRE, vancomycin-resistant enterococci; ICU, intensive care unit; APACHE, Acute Physiology and Chronic Health Evaluation score. ^b^VRE vs. controls, p = 0.03. ^c^VDE vs. VRE, p = 0.02. ^d^VDE vs. VRE, p = 0.03. ^e^VDE vs. controls, p < 0.01. ^f^VRE vs. controls, p < 0.01.

## Discussion

Vancomycin resistance is thought to be mediated primarily by the strain's acquiring the capacity to synthesize the cell wall by using D-alanine-D-lactate (*1*). In the first clinical reports of VDE infection, Fraimow et al. (*2*) and Green et al. (*4*) independently reported that D-alanyl-D-alanine supported the growth of a vancomycin-dependent *E. faecalis* strain (*2*) and a *vanB E. faecium* strain (*4*), respectively; Sng et al. (*9*) quantified the amount of D-alanyl-D-alanine required to support growth of their VDE strain. The phenomenon of vancomycin dependence may derive from the loss of a D-alanyl-D-alanine ligase in a VRE strain, which is then unable to survive unless vancomycin induces the production of D-alanine-D-lactate ligase (*2**,**4*). Previous reports (*2**–**12*) and our experience (Figure 2) suggest that infecting strains of VRE make the transition in situ to a state of vancomycin dependence only after prolonged exposure to vancomycin.

Sixteen patients infected by enterococci dependent on vancomycin for growth have been reported (*2**–**12*). In every case with data reported on prior antimicrobial exposure, the patients had also received a glycopeptide, vancomycin, or teicoplanin. We sought to minimize the effect of control group bias (*15*) by selecting as a control group concurrently hospitalized patients at risk for nosocomial infection with VRE or VDE but not infected with enterococci. We found that intense use of third-generation cephalosporins was the most important risk factor for both VDE and VRE when compared with the uninfected control group. This finding is in line with our recent observation of the striking commonality of risk factors for nosocomial colonization and infection with a diverse array of multiresistant pathogens, in particular, heavy exposure to third-generation cephalosporins (*16*). Selection pressure from broad-spectrum antimicrobial agents apears to promote nosocomial colonization with VRE, which, after prolonged exposure to vancomycin, may lead to the emergence of vancomycin dependence in the colonizing strain.

Renal insufficiency was the other risk factor identified in our study. The ecologic impact of vancomycin exposure is magnified and extended in patients with renal insufficiency, especially those with end-stage renal disease requiring hemodialysis (all 3 of our patients and 5 of the 16 previously reported cases), where a single dose persists in the patient's body for many days. The emergence of novel strains of *Staphylococcus aureus* exhibiting resistance to vancomycin has also been reported in this clinical setting (*17*). The prevalence of nosocomial infection or colonization with VDE can only be determined by the use of media containing vancomycin when processing cultures from patients at risk for VDE infection, namely those who have had prolonged exposure to vancomycin or third-generation cephalosporins, especially if they are already known to be colonized or infected by VRE.

These infections are clearly not trivial, although their clinical importance remains to be fully determined. Five of the 16 previously reported VDE infections were bacteremias (*4**,**7**,**9**,**11*). VDE was considered the immediate cause of death in one of our patients and a contributory cause in another. Green et al. (*4*) reported the spontaneous reversion of VDE to nondependence at 1 in 10^6^, which we confirmed in all three of our strains. Thus, vancomycin discontinuation alone may not be sufficient to treat patients with VDE infection, especially if the patient has renal failure.

The best management of infection with VDE—beyond source control and treatment with linezolid, quinupristin-dalfopristin, or daptomycin—remains to be determined. More effective antimicrobial stewardship policies are needed to prevent VDE, VRE, and other resistant nosocomial pathogens from emerging.
